# Plants in our combating strategies against *Mycobacterium tuberculosis*: progress made and obstacles met

**DOI:** 10.1080/13880209.2017.1309440

**Published:** 2017-04-07

**Authors:** Vivek Kumar Gupta, M. Madhan Kumar, Deepa Bisht, Anupam Kaushik

**Affiliations:** a Department of Biochemistry, National JALMA Institute for Leprosy & Other Mycobacterial Diseases (ICMR), Agra, India;; b Department of Immunology, National JALMA Institute for Leprosy & Other Mycobacterial Diseases (ICMR), Agra, India

**Keywords:** MDR, plant extracts, anti-TB phytomolecules, plant-based drugs

## Abstract

**Context:** Traditionally used plants for treating chest-related problems/tuberculosis (TB) have not been evaluated in detail and hence a thorough study is needed in this regard. This knowledge may find application in developing new anti-TB drugs.

**Objective:** This article elaborates on studying the activity of medicinal plants against different forms of *Mycobacterium tuberculosis* (*Mtb*) using different model strains, *in vitro* and *ex vivo* assays for studying the tuberculocidal activity and discusses the results from different studies on the activity against different forms of *Mtb* and human immunodeficiency virus-tuberculosis (HIV-TB) co-infection.

**Methods:** Scientific databases such as PubMed, Elsevier, Scopus, Google scholar, were used to retrieve the information from 86 research articles (published from 1994 to 2016) related to the topic of this review.

**Results:** Twenty-three plant species have been reported to possess active molecules against multi-drug resistant (MDR) isolates of *Mtb*. Seven plants were found to be active against intracellular *Mtb* and six against dormant bacilli. Seven plants were synergistically effective when combined with anti-TB drugs. Six studies suggest that the beneficial effects of plant extracts are due to their wide array of immuno-modulatory effects manifested by the higher expression of cytokines. Some studies have also shown the dual activity (anti-HIV and anti-TB) of plants.

**Conclusion:** We emphasize on identifying plants based on traditional uses and testing their extracts/phytomolecules against MDR strains, intracellular *Mtb* as well as against dormant *Mtb*. This will help in future to shorten the current therapeutic regimens for TB and also for treating HIV-TB co-infection.

## Introduction

*Mycobacterium tuberculosis* (*Mtb*), a highly infectious bacterial pathogen and causative agent of tuberculosis (TB) infects about one third of the world’s population. In the year 2015 there were 1.4 million deaths due to TB and an estimated 0.4 million people who died due to HIV-TB coinfection. The six countries that stand out as having the largest number of incident cases (60%) in 2015 were India, Indonesia, China, Nigeria, Pakistan, and South Africa (World Health Organization 2016a).

The treatment of TB started 73 years ago since the discovery of streptomycin and after that many drugs were introduced, despite that TB remains as one of the leading infectious diseases worldwide. TB infection occurs by engulfment of bacterium by alveolar macrophages, where bacilli evade killing and continue to multiply by avoiding phagosome-lysosome fusion. Additional macrophages and other immune cells then become localized to the site of infection creating an ordered cellular architecture known as granuloma (Barry et al. 2009).

In the granuloma, although actively replicating bacilli are found, non-replicating persistent (NRP) (dormant) form of *Mtb* can also be found which are induced by the environmental conditions (found in specific granuloma types, particularly those associated with hypoxia/anoxia, nutrient deprivation and nitric oxide production). The NRP state of *Mtb* is characterized by the presence of non-dividing bacilli with low-metabolic state and also resistance to standard anti-TB agents. Anti-TB drugs which are also able to kill bacilli inside the environment of granuloma are likely to offer the best opportunity to reduce treatment length and eliminate relapse (Lin & Flynn 2010).

As with other bacterial diseases, it is expected that drug resistance occurs almost immediately after a new chemo-therapeutic agent is introduced into the market. *Mtb* strains which are resistant to at least isoniazid and rifampin are termed multi drug resistant (MDR) (Caminero et al. 2010). In the past few years, *Mtb* strains resistant to isoniazid, rifampin, fluoroquinolones and one of the second-line injectable agents have been discovered and such strains have been defined as extensively drug resistant (XDR) (Zhang & Yew 2015).

About 480,000 people developed MDR-TB and 100,000 developed rifampin resistant TB (RR-TB) globally in 2015. It was estimated that 3.9% of new cases and 21% of previously treated cases were having MDR-TB or RR-TB. India, China and the Russian federation accounted for 45% of the total of 580,000 cases. XDR-TB has been reported by 117 countries for the year 2015. On an average, an estimated 9.5% of people with MDR-TB have XDR-TB (World Health Organization 2016b). Treatment of drug-resistant TB is more expensive and time-consuming. The cure rates for MDR-TB are also lower, typically ranging from 50% to 70% (Koul et al. 2011). Current anti-TB drugs are not very efficient in killing the dormant as well as intracellular forms of *Mtb*.

TB is a leading killer of HIV-infected individuals leading to 1 in 3 HIV deaths due to TB (World Health Organization 2016a). The association of TB with HIV and the increasing emergence of MDR and XDR-TB have worsened the situation and posed a serious health threat. Rifampin, induces cytochrome-P450 enzymes in patients having HIV-TB co-infection, leading to a reduction in systemic exposure of some commonly used anti-retroviral agents (Gandhi et al. 2010).

Keeping the aforesaid facts in view, new and effective alternatives for the treatment of TB are urgently needed and in this context, medicinal plants may represent a potential option to combat the threat of TB. This review elaborates on different medicinal plants which have been tested for treating latent to drug-resistant forms of *Mtb* as well as their use in treating HIV-TB co-infection. As the current armamentarium against *Mtb* suffers from demerits ranging from toxicity to extensive therapy, the unexplored plants with killer potential will offer solutions and will further add significance to TB treatment. This review also underlines the importance of medicinal plants in TB treatment as much of the work on their efficacy in TB is undermined or little known. The knowledge gained regarding the use of medicinal plants in TB and the promising results obtained from earlier studies warrant their use as an immunomodulator or in using them as a supplement to currently used anti-TB drugs.

## Need for exploring plants with anti-TB activity

In recent years, there has been an increasing interest in natural product based medicines from plant origin. The plants are an important source of biologically active secondary metabolites which have enormous therapeutic potential. World Health Organization (WHO) estimates that about 80% of population living in Africa, and 40% of the population from China are using plant based traditional medicines (TM) for their primary health care needs. In many countries, TM continues to be widely used, even though allopathic medicine is often readily available (World Health Organization 2002-2005). The medicinal plants are the backbone of TM and around 3300 million people in the under developed countries utilize medicinal plants on a regular basis (Pan et al. 2013). The medicinal plants used in traditional medicine by various traditional/tribal healers and their knowledge about medicinal uses of plants which passes verbally from generation to generation without any documentation are also important criteria for plant based drug discovery program. New anti-TB drug candidates are urgently required to ensure the generation of novel treatment regimens. Use of unexplored sources of plants and lead optimization strategies may also improve the efficiency of future anti-TB drug discovery.

## Criteria for selection of plants

Among 420,000 plant species reported worldwide, very few plants have been screened for biological activity (Pan et al. 2013). So, there are tremendous scopes to identify the unexplored plants for biological activity. Selection of the plants for biological activity is the first and important step in drug discovery program. The various selection approaches are based on random, ethno-botanical, ethno-pharmacological, traditional uses and zoo-pharmacognosy (Huffman 2003). Several authors have suggested that the ethno-based approach is more efficient over random selection method for plants having therapeutic potential (Rios & Recio 2005).

## Extraction of secondary metabolites from plants

Plants contain a variety of secondary metabolites. So, a standard extraction scheme is required for extraction of various group of secondary metabolites for the evaluation of biological activity. Plant extracts are prepared by maceration or percolation of fresh green plants or dried powdered plant material in water and/or organic solvents (Eloff 1998). It is also important to consider the traditional approaches to prepare the extract as described by the traditional healer to mimic the traditional herbal drug as closely as possible.

## Selecting model strains for studying anti-TB activity of plants

*Mycobacterium tuberculosis* H_37_Rv (ATCC 27294) is the widely used target strain for evaluation of anti-TB activity (Pauli et al. 2009). This strain has a drug susceptibility profile fairly representative of most drug susceptible clinical isolates. In the initial stage of plant based drug discovery, it is not possible to test each of the extract against this bacterial strain due to its highly infectious nature, slow growth and the complex infrastructure needed. These features make this option difficult in many laboratories where biosafety level (BSL)-3 facilities and complex infrastructure are not available. So, the screening concept is usually validated first in fast-growing, surrogate, nonpathogenic *Mycobacterium* species and subsequently confirmed in *Mtb*.

To avoid the use of *Mtb,* fast-growing and nonpathogenic *Mycobacterium* species which can be handled under BSL-2 serve as surrogate model organisms (Table 1). These provide a rapid, less hazardous and more economic platform for the primary screening of plant extracts/phytomolecules. Most of the researchers have evaluated plant extracts/fractions/pure compounds against *M*. *aurum and M. smegmatis* as a surrogate for the highly pathogenic *Mtb* in anti-TB primary screening due to their fast growth rate, sharing similarity in cell wall mycolic acid and antibiotic susceptibility profiles (Chaturvedi et al. 2007; Altaf et al. 2010; Gupta & Bhakta 2012; Phelan et al. 2015).

**Table 1.** Model strains of *Mycobacterium* spp. for primary screening of plant anti-TB activity (Chaturvedi et al. 2007; Altaf et al. 2010; Gupta & Bhakta 2012; Phelan et al. 2015).

Model strains	General features	Important properties
*M. smegmatis*	Fast growing, saprophyte, generation time 2–3 h., temperature range 22–45 °C.	Naturally resistant towards isoniazid and rifampin and have a profile similar to MDR strains of *Mtb*.
*M. phlei*	Fast-growing, saprophyte, temperature range 22–52 °C.	Short rods, acid fast in young cultures but staining irregularly after incubation for 5–7 days.
*M. marinum*	Causes a systemic TB-like disease in a large number of poikilothermic animals, growth optimum of 33–35 °C, generation time ∼4 h.	It is easily manipulated on the laboratory bench top under BSL-2 safeguards.
*M. fortuitum*	Rapidly growing, saprophyte, infrequent human pathogen,generation time 4–5 h, growth optimum of 30–37 °C.	Like *Mtb*, it resides intracellularly in vacuoles restrictinginterferon-γ-induced nitric oxide production and limits thematuration of phagosomes.
*M. aurum*	Environmental bacteria typically found in damp conditionsfast-growing, doubling time of 2–3 h.	Possesses a similar cell wall composition to *Mtb*, as well assharing intracellular therapeutic targets, gene organization and drug susceptibility.

## *In vitro* and *ex vivo* anti-TB assays for evaluating the plant extracts/compounds

Different *in vitro* methods for assessing tuberculocidal activity of plant extracts include classical agar diffusion, micro and macro broth dilution assays, radiometric BACTEC 460, MGIT 960 reporter gene assays (Table 2).

**Table 2.** Bioassays for evaluating the plant extracts/compounds against different forms of *Mtb*.

Name of the assay	Principle	Advantages/disadvantages	References
(A) Activity against replicating *Mtb*			
Agar diffusion	Diffusion of test compounds on the surface of medium containing agar-agar.	Rapid screening of plant extracts/compounds against non pathogenic model strains.	(Gupta et al. 2011)
LJ proportion method	Incorporation of the test sample in LJ medium at different concentration (v/v per cent).	The major disadvantage is the requirement for at least 18 days to detect growth.	(Gupta et al. 2010)
Micro-broth dilution using oxidation/reduction indicator	MIC determined using oxidation/reduction indicator dyes (resazurin, alamar blue, tetrazolium) which makes this a more rapid and sensitive assay.	Commonly used 96-well microplate assay which offers advantage of small sample requirements, low cost and HTS.	(Martin et al. 2003; Navarro-García et al. 2011; Kumar et al. 2013; O'Neill et al. 2014)
Radiometric BACTEC 460 assay	Radiometric automated system which involves the measurement of ^14^CO_2_ produced by *Mtb* growing in broth containing ^14^C-labeled palmitic acid.	HTS assay and result can be observed within 7–10 days. The main disadvantage of this assay is the cost.	(Gupta et al. 2011)
BACTEC MGIT 960 assay	Non-radiometeric automated system that uses modified Middlebrook 7H9 broth which contains a fluorescent sensor ruthenium chloride pentahydrate.	It is an excellent, high throughput method able to provide rapid and reliable results within 7–10 days.	(Jyoti et al. 2016)
Reporter gene assays	Based on the use of reporter genes (red fluorescent protein, green fluorescent protein, Luciferase).	Inexpensive to be used for routine HTS of anti-TB compounds with limited commercial application.	(Collins et al. 1998; Songsri & Nuntawong 2016)
B) Activity against dormant bacteria			
Wayne’s hypoxia model	Based on the induction of hypoxic conditions with constant shaking for a defined period of time (usually 24 days).	Although in this model, hypoxia-induced latent bacilli were found to be sensitive for metronidazole but only one stress factor was introduced.	(Wayne & Sramek 1994)
Betts starvation model	A nutrient starvation model was developed by transferring bacilli into nutrient deficient medium and incubated at 37 °C for 6 weeks.	Model simulates *Mtb* in physiologically dormant states but only one stress factor was introduced.	(Betts et al. 2002)
LORA assay	For screening of compounds against non-replicating *Mtb* using an *Mtb* pFCAluxAB strain.	High-throughput luminescence-based assay to screen antimicrobial agents against NRP form of *Mtb*.	(Cho et al. 2007; Elkington et al. 2014)
Multiple stress model	Applying combined stresses of low oxygen (5%), high CO_2_ (10%), low nutrient and acidic pH (5.0).	This model efficiently generates *bacilli* meeting all criteria of dormancy, and this multiple stress method is adaptable to HTS.	(Deb et al. 2009)
C) Intracellular sterilizing activity inside macrophages			
*Ex vivo* intra cellular activity	Macrophages (THP-1 cells, J774 cells) infected with a multiplicity of infection (MOI) of 10.	Bacterial viability assessed by colony forming unit enumeration.	(Jiménez-Arellanes et al. 2013; Luo et al. 2013)

*Ex-vivo* assays (using mouse/human macrophages) are used for determining the intracellular *Mtb* killing or sterilizing activity of test compounds. In most cases, macrophages are infected with *Mtb* and are then treated for 3–7 days with test compounds for detecting intracellular anti-TB activity (Jiménez-Arellanes et al. 2013; Luo et al. 2013).

The latent form of tubercle bacilli also presents a major obstacle in anti-TB therapy. The first *in vitro* model for studying dormant bacilli was put forth by Wayne and Sramek (1994). In the Wayne model, *Mtb* is grown with constant shaking for a defined period of time (usually 24 days) which leads to hypoxic conditions (Wayne & Hayes 1996). This model has been used by various researchers for genomic and proteomic studies of *Mtb* under dormancy but to our knowledge, it has not been applied for screening of anti-TB activity of plants. This is due to the difficulty in rapid screening of compounds in this model. A high-throughput, luminescence-based low oxygen-recovery assay (LORA) is useful in identification of plant extracts/compounds active against non-replicating, dormant *Mtb* (Cho et al. 2007). In the LORA model, *Mtb* H_37_Rv contains a plasmid with an acetamidase promoter which drives a bacterial luciferase gene and is adapted to low-oxygen conditions as established in Wayne's model by extended culture in a fermentor with a 0.5 headspace ratio. Although LORA is a high-throughput screening (HTS) assay and is in use for evaluating the activity of plant extracts/compounds against NRP bacilli, it includes only one stress factor which does not fulfill all the criteria of dormancy.

In a granuloma, *Mtb* shuts down some of the metabolic pathways to economize the energy in cells due to nutrient deprivation. Based on this phenomenon, another dormancy model was developed by Betts et al. (2002) which is a nutrient starvation model in which bacilli are transferred into phosphate buffered saline and incubated at 37 °C for 6 weeks. During this period, the mycobacteria are shifted from active to the latent state. This model is used only for gene and protein expression studies of NRP but anti-TB activity of plant extracts has not been evaluated using this model. Deb et al. (2009) developed a multiple stress model by applying combined stresses of low oxygen (5%), high CO_2_ (10%), low nutrient and acidic pH (5.0). The degree of antibiotic resistance in *Mtb* NRP in this model under multiple-stress conditions is significantly higher than that obtained in the Wayne’s hypoxia model. This model efficiently generates *bacilli* meeting all criteria of dormancy but needs validation for drug candidature testing and needs to be feasible for HTS.

Although various methods are reported for screening of plant extracts against replicating bacilli and are validated, very few assays are available for evaluating the plant extracts against dormant NRP of *Mtb*. Very few plants/compounds have been explored for anti-TB activity against dormant NRP form of *Mtb*. It is important to identify many plants/plant-derived compounds, develop and validate a new screening system for determining the inhibitors of latent TB bacilli.

## Criteria for defining antimicrobial potential of plant extracts and compounds

In most of the *in vitro* assays, the activity of extracts/fractions/compounds is generally expressed in terms of minimum inhibitory concentration/inhibitory concentration (MIC/IC). Significant activity relates to IC_50_ values below 0.1 mg/mL for plant extracts and 0.025 mg/mL for pure compounds (Silva et al. 2013). MIC higher than 1 mg/mL for extracts or 0.1 mg/mL for isolated compounds should be avoided, whereas remarkable antimicrobial activity can be considered in case of concentrations below 0.1 mg/mL for extracts and 0.01 mg/mL for pure compounds (Rios & Recio 2005).

## Search for anti-tuberculosis potential in plants

To achieve complete cure, it is expected that a drug should kill all forms of *Mtb* that exist in clinical TB (whether extracellular or intracellular and replicating or non-replicating (dormant) mycobacteria). In addition, phytomolecules should have different mechanisms of action to ensure effectiveness against strains that are resistant to standard anti-TB drugs.

## Plants active against MDR strains of *Mtb*

In the scientific literature, several papers describe the extracts and the pure compounds obtained from medicinal plants that exhibit significant *in vitro* activity against *Mtb* H37Rv (Gautam et al. 2007; Santhosh & Suriyanarayanan 2014) but only a small number of plants have been evaluated for anti-TB activity against drug-resistant strains. So, it is very important to identify plants active against *Mtb* strains resistant to standard anti-TB drugs for plant-based drug discovery.

Jimenez-Arellanes et al. (2003) identified anti-TB activity in traditionally used plants for treating respiratory diseases. Among the active plants, *Lantana hispida* Kunth (Verbenaceae) showed activity against different monoresistant *Mtb* strains. Isolated dihydro-β-agarofuran sesquiterpenes (1α-acetoxy-6β,9β-dibenzoyloxy-dihydro-β-agarofuran) from the leaves of *Celastrus vulcanicola* Donn. Sm. (Celastraceae) exhibited anti-TB activity against the MDR strain with a MIC value of 0.0062 mg/mL, comparable to or better than isoniazid or rifampin, two of the best first-line drugs commonly used in the treatment of TB (Torres-Romero et al. 2011).

Phytomolecule plumericin isolated from *Plumeria bicolor* Ruiz & Pav. (Apocynaceae) showed activity against sensitive as well as four MDR strains of *Mtb* with MIC values of 0.0015 to 0.002 mg/mL and MBC (minimum bactericidal concentration) values of 0.003–0.004 mg/mL. Active compound showed an advantage over rifampin (80 times) and isoniazid (8 times) by being highly active against the MDR strains (Kumar et al. 2013).

Naphthoquinones, plumbagin and its dimers maritinone and 3,3′-biplumbagin from *Diospyros anisandra* S.F.Blake (Ebenaceae) showed the strongest activity against *Mtb* strains (MIC 0.0015–0.0033 mg/mL). The bioactivity of maritinone and 3,3′-biplumbagin were 32 times more potent than rifampin against the pan-resistant strain, and both dimers were shown to be nontoxic (Uc-Cachón et al. 2014). List of the various plants and their active phytomolecules against MDR *Mtb* strains are summarized in Table 3.

**Table 3.** Plants active against MDR strains of *Mtb*.

Plants (Family)	Traditional uses	Active phytomolecules/fractions	MIC against MDR-*Mtb*	References
*Aristolochia taliscana* Hook and Arn. (Aristolochiaceae)	Coughs and respiratory infections	Licarin A & B; eupomatenoid-7	0.0125–0.050 mg/mL	(León-Díaz et al. 2010)
*Aristolochia brevipes* Benth. (Aristolochiaceae)	Arthritis, diarrhea, cough with blood	Aristolactam I	0.0125–0.025 mg/mL	(Navarro-García et al. 2011)
*Aristolochia elegans* Mast. (Aristolochiaceae)	Diarrhea, fever	Fargesin; (8R,8´R,9R)-cubebin	0.025–0.050 mg/mL	(Jiménez-Arellanes et al. 2012)
*Artemisia capillaris* Thunb. (Asteraceae)	Malaria	Ursolic acid, hydroquinone	0.0125–0.025 mg/mL	(Jyoti et al. 2016)
*Azorella* compacta Phil.*, A. madreporica* Clos. (Apiaceae)	Asthma, bronchitis	Azorellanes; azorellanol	0.0125 mg/mL	(Molina-Salinas et al. 2010)
*Beilschmiedia tsangii* Merr. (Lauraceae)	No known traditional uses	Beilschmin A	0.0025 mg/mL	(Chen et al. 2007)
*Blepharodon nitidum* (Vell.) J.F. Macbr. (Asclepiadaceae)	No known traditional uses	25-Hydroperoxycycloart-23-en-3β-ol	0.0125 mg/mL	(Aponte et al. 2008)
*Celastrus vulcanicola* Donn. Sm. (Celastraceae)	No known traditional uses	1α-Acetoxy-6β, 9β-dibenzoyloxy-dihydro-β-agarofuran	0.0062 mg/mL	(Torres-Romero et al. 2011)
*Chamaedorea tepejilote* Liebm. (Palmae)	Source of traditional food	Ursolic acid	0.025 mg/mL	(Jimenez-Arellanes et al. 2005)
*Citrullus colocynthis* (L.) Schrad. (Cucurbitaceae)	TB and other respiratory diseases	Ursolic acid, cucurbitacin E2-0-β-d-glucopyranoside	0.050–0.125 mg/mL	(Mehta et al. 2013)
Clavija procera B.Ståhl (Theophrastaceae)	Snake bite	Oleanane triterpenoid aegicerin	0.0016–0.00312 mg/mL	(Rojas et al. 2006)
*Curcuma longa* L.(Zingiberaceae)	Whooping cough	Isoxazole analogs of curcuminoids	0.0019–0.00312 mg/mL	(Changtam et al. 2010)
*Euclea natalensis* A.DC. (Ebenaceae)	Bronchitis	Diospyrin	0.1 mg/mL	(Lall & Meyer 2001)
*Foeniculum vulgare* Mill. (Apiaceae)	Digestive problems	5-Hydroxy furanocoumarin	0.100–0.200 mg/mL	(Esquivel-Ferriño et al. 2012)
*Justicia adhatoda* L. (Acanthaceae)	Colds, cough, and other respiratory disorders	Vasicine acetate; 2-acetylben-zylamine	0.2 mg/mL	(Ignacimuthu & Shanmugam 2010)
*Kaempferia galangal* L. (Zingiberaceae)	Cold, cough	Ethyl *p*-methoxycinnamate	0.242–0.485 mM	(Lakshmanan et al. 2011)
*Lantana hispida* Kunth (Verbenaceae)	Bronchitis, cough	Oleanolic acid	0.025–0.050 mg/mL	(Jimenez-Arellanes et al. 2007)
*Larrea tridentata* Coville. (Zygophyllaceae)	Fever, colds, respiratory infections	Dihydroguaiaretic acid;4-epi-larreatricin	0.0125–0.050 mg/mL	(Favela-Hernández et al. 2012)
		5,4′-dihydroxy-3,7,8,3′-tetramethoxy flavone	0.025–0.050 mg/mL	
*Plectranthus grandidentatus* Gurke (Lamiaceae)	No known traditional uses	Abietane and its derivatives	0.0031–0.0039 mg/mL	(Rijo et al. 2010)
*Plumeria bicolor* Ruiz & Pav. (Apocynaceae)	Rheumatism, diarrhea	Plumericin	0.0015–0.002 mg/mL	(Kumar et al. 2013)
*Tabernaemontana elegans* Stapf. (Apocynaceae)	Edible plant used in Thai cuisine	Tiliacorinine, 2′-nortiliacorinine, tiliacorie	0.0031 mg/mL	(Sureram et al. 2012)
*Diospyros anisandra* S.F.Blake (Ebenacea)	Cutaneous disorders	Plumbagin	0.0015 mg/mL	(Uc-Cachón et al. 2014)
		Maritinone, 3,3′-biplumbagin	0.0033 mg/mL	

## Intracellular *Mtb* killing by plants

A drug is said to be very efficient if it is rapidly bactericidal and also possess potent sterilizing activity, i.e., killing even the intracellular forms of pathogen at a shorter time period. In this regard, plants can be explored and identified for anti-TB intracellular sterilizing activity inside macrophages that could shorten current therapeutic regimens. In literature, intracellular *Mtb* killing by plant extracts have been investigated by many studies as detailed below:

The compound 7-methyljuglone isolated from the roots of *Euclea natalensis* A.DC. (Ebenaceae) exhibited activity against intracellular *Mtb* Erdman strain within J774.1 macrophages at a concentration of 0.57 μg mL^−1^. The intracellular activity of this compound was better when compared to anti-TB drugs streptomycin and ethambutol (Lall et al. 2005).

Green tea polyphenol (9-epigallocatechin-3-gallate) was found to inhibit *Mtb* survival within human macrophages (Anand et al. 2006). The down-regulation of host molecule tryptophan-aspartate containing coat protein (TACO) gene expression by epigallocatechin-3-gallate was accompanied by inhibition of *Mtb* survival within macrophages as assessed by flow cytometry and colony counts.

*Pelargonium reniforme* Curtis (Geraniaceae) and *P. Sidoides* DC. (Geraniaceae) are used by native populations in southern Africa as folklore medicine to treat respiratory tract infections. Their root extract constituents myricetin and quercitin-3-O-β-D-glucoside were shown to kill *Mtb* inside mice peritoneal macrophages with significant reduction in intracellular viability (at the 99% confidence level) only at the highest concentration tested (0.250 mg/mL) (Kim et al. 2009).

An alkaloid decarine from *Zanthoxylum capense* (Thunb.) Harv. (Rutaceae) was found to be active against intracellular *Mtb* in THP-1 macrophage infection model at concentrations of 0.0062–0.025 mg/mL which was superior to that of pyrazinamide at 0.2 mg/mL (Luo et al. 2013).

Two active compounds ursolic acid (UA) and oleanolic acid (OA) were isolated from the medicinal plants *Chamaedorea tepejilote* Liebm. (Arecaceae) and *Lantana hispida* Kunth (Verbenaceae) (used in Mexican traditional medicine) for the treatment of respiratory complaints such as cough, bronchitis, colds and pneumonia (Jiménez-Arellanes et al. 2013). These compounds exhibited intracellular anti-TB activity in *Mtb* infected macrophage cell line J774A. The combination of UA and OA were found to be more effective in the killing of intracellular *Mtb*.

Although few plants have been studied for their intracellular *Mtb* killing ability, still many plants can be explored for this potential.

## Activity of plants against dormant bacilli

Targeting dormant, non-replicating *Mtb* is also very important for complete cure latent TB infection (Fattorini et al. 2013). In this regard, very limited number of plants have been explored for this activity.

Investigation of *n*-hexane extract of the aerial parts and roots of *Juniperus communis* L. (Cuppressaceae) yields antimycobacterial terpenoids longifolene and totarol (Gordien et al. 2009). Totarol manifests the best activity against *Mtb* H37Rv (MIC of 73.7 μM). It has also been shown to be active against isoniazid, streptomycin and moxifloxacin resistant variants. Totarol also exhibited activity against the subpopulation of *Mtb* in the NRP state using a low oxygen recovery assay (LORA) (MIC of 81.3 μM). The cytotoxicity studies indicate that the isolated compound was relatively less toxic towards mammalian cells.

*Alpinia galanga* (L.) Wild. (Zingiberaceae) extensively used in diets as well as in the traditional systems of medicine (Thai, Ayurveda, Unani, and Chinese) exhibits anti-TB activity under reducing oxygen concentrations using candle jar method against dormant and non-replicating bacteria. The acetone and ethanolic extracts of these plants has also showed intracellular anti-TB activity inside human lung epithelial carcinoma cell line (Gupta et al. 2014). *Marsypopetalum modestum* Xue and R.M.K. Saunders (Annonaceae) is traditionally used by people of Laos to treat symptoms of TB. Investigation of the phytochemical constituents of *M. modestum* by Elkington et al. (2014) led to the isolation of dipyrithione, which was shown to exhibit activity against dormant tubercle bacilli through LORA.

In order to maintain *Mtb* viability in dormancy, glyoxylate enzymes get upregulated in order to continue generating energy from an alternative carbon source, namely, lipids. The glyoxylate cycle is activated in *Mtb* growing on fatty acids as exclusive carbon source, and also during chronic infection in mice suggesting that lipids are accessible as nutrients *in vivo* (McKinney et al. 2000). Isocitrate lyase (ICL) was identified as gate enzyme of the glyoxylate shunt, a short-cut of the tricarboxylic acid (TCA) cycle, by passing steps of carbon loss by CO_2_ formation. The potential of ICL as a drug target has been evaluated in many studies as discussed in a review by Lee et al. (2015).

Bai et al. (2006) targeted ICL in *Mtb* using plant extracts. A total of 465 traditional Chinese medicines were screened against *Mtb* ICL. The extracts of *Zingiber officinale* Rosc. (Zingiberaceae) (IC_50_ of 0.0477 mg/mL) and *Illicium verum* Hook. f. (Schisandraceae) (IC_50_ of 0.0182 mg/mL), were reported to be active. In another study by Liang et al. (2011), chelerythrine extract from the *Chelidonium majus* L. (Papaveraceae) was also reported as a potential drug which causes five-fold decrease in ICL gene expression. Although many authors have targeted ICL directly, they have not studied the ICL inhibition of dormant *Mtb* under *in vitro* stress environment.

## Synergistic anti-TB potential of plants

Synergistic combination therapy is a possible approach to overcome drug resistance. The synergistic combination offers several potential advantages (1) efficient bactericidal effect (2) less chances to develop resistance against combination (3) more efficiency in killing the drug resistant strains and better tolerability (Greco et al. 1995). The synergistic interaction can also be explored for killing drug resistant strains of *Mtb* as well as different forms of bacilli in the host. *In vitro* synergistic effects resulting from the combination of antibiotics with various plant compounds has been studied by many authors. Some phytomolecules were found to be synergistically effective in combination with rifampin, isoniazid, ethambutol. The 4- to 16-fold reduction in MIC of anti-TB drugs were reported in the presence of phytomolecules (Table 4). The synergistic interaction can also be explored for killing drug resistant strains of *Mtb* as well as different form of bacilli in the host. Synergistic effects resulting from the combination of antibiotics with various plant extracts has been studied earlier (Table 4).

**Table 4.** List of plants having synergistic activity with anti-TB drugs.

Plants (Family)	Phytomolecules	Synergistic interactionwith anti-TB drugs	Fold reduction inMIC of anti-TB drug	FIC index^a^	References
Commercial source (plant origin)	Oleanolic acid	Isoniazid	4 to 16	0.121–0.347	(Ge et al. 2010)
		Rifampicin	8 to 16	0.113–0.168	
		Ethambutol	4 to 16	0.093–0.266	
*Galenia africana* L. (Aizoaceae)	(2S)-5,7,2′-Trihydroxyflavonone	Isoniazid	16	0.12	(Mativandlela et al. 2009)
	(E)-3,2′,4′-Trihydroxy-3′-methoxychalcone	Isoniazid	4	0.5	
*Euclea natalensis* A.DC. (Ebenaceae)	7-Methyljuglone	Rifampicin	4 to 6	0.5	(Bapela et al. 2006)
		Isoniazid	4 to 6	0.2	
*Notopterygium incisum* K.C.Ting ex H.T.Chang (Apiaceae)	Isoimperatorin	Rifampicin	5 to 20	0·133–0·472	(Guo et al. 2014)
		Isoniazid		0·123–0·475	
		Ethambutol		0·124–0·25	
*Piper nigrum* L.(Piperaceae)	Piperine	Rifampicin	4 to 8	<0.5	(Sharma et al. 2010)
*Knowltonia vesicatoria* (L.f.) Sims (Ranunculaceae)	Plant extract	Isoniazid	8	0.25	(Labuschagné et al. 2012)

a Synergy generally defined by FIC index values of ≤0.5.

Most of the researchers have applied checkerboard synergy assay for synergistic studies. Synergy is generally defined by fractional inhibitory concentration index (FICI) values of ≤0.5, antagonism by FICI values of >4.0, and no interaction by FICI values from 0.5 to 4.0 (Odds 2003).

## Immunomodulatory activity of plants in *Mtb* infection

There is a growing interest to use herbal medicines as multi-component agents to modulate the complex immune system in the prevention of infections rather than treating immune-related diseases. Many therapeutic effects of plant extracts have been suggested to be due to their wide array of immunomodulatory effects and influence of the immune system on the human body. The Ukrainian multi-herbal dietary supplement Dzherelo (Immunoxel) contains water-alcohol extracts of 27 medicinal *plants* which have been shown to have a favourable effect on the immune status and viral burden in HIV/TB patients when given as an immunomodulating adjunct to anti-TB therapy (Nikolaeva et al. 2008). The immuno-modulatory effect of Dzherelo has been evaluated in a 60 days trial in 75 newly diagnosed TB patients. Liver damage indicators were found to be decreased to normal levels and kidney failure markers were normalized in Dzherelo recipients in groups treated with isoniazid, rifampin, pyrazinamide and streptomycin (HRZS) in comparison to group treated only with HRZS. Radiological recovery was significant and mycobacterial clearance was higher in Dzherelo recipients in groups treated with HRZS in comparison to group who were treated only with HRZS (Zaitzeva et al. 2009). Dzherelo has also shown immunomodulatory effects in patients with XDR-TB infection (Prihoda et al. 2009).

Anti-TB activity of UA and OA has been evaluated in BALB/c mice infected with *Mtb*. Animals which were treated with UA and OA showed a higher expression of IFN-γ and TNF-α in the lungs than control animals (Jiménez-Arellanes et al. 2013). Sharma et al. (2014) evaluated the immunomodulatory activity of piperine in *in vitro* and *in vivo* models. Piperine enhanced the efficacy of rifampin in a murine model of *Mtb* infection. Piperine induced proliferation of T and B cells, increased Th-1 cytokines and enhanced macrophage activation in *ex-vivo* murine splenocytes. In an *in vivo Mtb* mice infection model, piperine activated the differentiation of T cells into Th-1 sub-population (CD4+/CD8 + subsets) with an increase in secretion of Th-1 cytokines (IFN-γ and IL-2) by these cells. The efficacy of piperine has been evaluated in a multicentric clinical trial conducted across India in patients with radiologically confirmed diagnosis of pulmonary TB (Chawla 2010). Recently, Bai et al. (2016) evaluated immune-modulatory effects of polyphenol curcumin from *Curcuma longa* L. (Zingiberaceae) using macrophage infection model. Curcumin was found to protect against *Mtb* infection in human macrophages. They observed that curcumin was an inducer of caspase-3-dependent apoptosis and autophagy in *Mtb*-infected macrophages.

## Search for plants with anti-TB and anti-HIV activity

In HIV positive people, there is a 20–30 fold increased relative risk of developing TB disease from latent state when compared to those without HIV (Pawlowski et al. 2012). In 2015, around 0.4 million deaths occurred due to HIV-TB confection (World Health Organization 2016a). The treatment of patients co-infected with TB/HIV presents also additional challenges such as drug intolerance and drug–drug interaction. It will be a great achievement to discover anti-TB drugs also possessing anti-HIV potential or vice versa.

Researchers have also evaluated plants for dual activity against HIV and *Mtb*. Medicinal plants have been reported to display anti-HIV-1 activity (Helfer et al. 2014). (+)-Calanolide A, isolated originally from the rainforest tree *Calophyllum lanigerum* Miq. (Calophyllaceae) as an anti-HIV agent, has been found also to be active against *Mtb* (MIC 0.0031 mg/mL) and a range of drug-resistant strains (MIC 0.008–0.016 mg/mL) (Xu et al. 2004). The extracts from *Annona muricata L. (*Annonaceae*)* and *Artemisia afra* Jacq. Ex Willd (Asteraceae) have shown both anti-TB and anti-HIV activity. *Annona muricata* ethanolic extract exhibited anti-TB activity with a MIC of 0.125 mg/mL. *Artemisia afra* aqueous extract showed weak HIV-1 reverse transcriptase (HIV-RT) inhibition while the ethanol extracts of *A. afra* and *A. muricata* showed inhibition of HIV-1 integrase with an IC_50_ < 100 μg/mL (van de Venter et al. 2014). These extracts could be an important source of compounds for treating HIV-TB co-infection.

The three plant extracts from *Amphipterygium glaucum* Hemsl. & Rose ex Standl. (Anacardiaceae), *A. molle* Hemsl. & Rose ex Standl. and *A. simplicifolium* (Standl.) Cuevas-Figueroa fairly inhibited *Mtb* with an IC_50_ of 0.0018–0.0023 mg/mL, but their IC_50_ against HIV-RT was 0.0592–0.0978 mg/mL. Effect of the extracts from *Calophyllum brasiliense* Cambess. (Calophyllaceae), *Vismia baccifera* (L.) Planch. & Triana (Hypericaceae), and *V. mexicana* on *Mtb* was noteworthy (IC_50_ 0.003–0.0364 mg/mL) which also inhibited HIV-RT (IC_50_ 0.0262–0.0351 mg/mL) (Gómez-Cansino et al. 2015).

## Conclusions and future perspective

Drug resistant-TB and HIV-TB co-infection have become a great threat to global health. Targeting tubercle bacilli is always a challenge. The current anti-TB drugs are less effective against MDR and XDR strains. So, considering the adverse situation, the search for new effective drugs against TB is now more important.

Natural products derived from medicinal plants may play a critical role in anti-TB drug discovery. Recently, advances have been made in speeding up the discovery of novel TB drugs including diversifying strategies for plant selection, HTS of plants and natural product chemistry.

This review has outlined the strategy for targeting the tubercle bacilli using plants as therapeutic agents including selection of plants, selection of model strain for primary screening, different anti-TB bioassays, plant extracts/phytomolecules active against MDR strains, sterilizing activity, activity against dormant NRP bacilli, synergistic activity with current anti-TB drugs and plants having dual activity in HIV-TB co-infection.

Most of the plants evaluated in the above said studies exhibited *in vitro* activity against *Mtb* and they are known for their medicinal uses mainly in respiratory problems, cough, cold, fever and chest complaints which shows the importance of tradition-based plant selection. This type of selection is always important for drug discovery and has less possibility to possess toxicity, because these plants have already been used in traditional medicine with no/or fewer side effects. Traditional herbalists prescribing plant-based treatments have for a long time played an important role in the provision of primary healthcare, especially in rural areas where most of the population is poor and unable to afford modern drugs.

Some of the plants (refer Table 3) are remarkably important because they are highly effective against MDR *Mtb* strains at very low concentrations (0.001 to 0.005 mg/mL) and also exhibit selective toxicity. There is also a good opportunity to explore the plants having synergistic activity with standard anti-TB drugs. These phytomolecules acting synergistically may enhance the bactericidal action of current anti-TB drugs at a lower concentration. There is also a need to identify the phytomolecules having novel mechanism of action to overcome the drug resistance problem. Very few plants have been explored for anti-TB activity against NRP form of bacilli under dormant conditions. So, there is a tremendous scope to identify the plants having activity against drug tolerant, dormant, NRP form of *Mtb* bacilli. Phytomolecule (+)-calanolide A is a good example of a single molecule that can target both *Mtb* and HIV in HIV-TB co-infection. This identification of dual activity against both HIV and *Mtb* is unique and opens a new chapter in drug discovery in this area.

Although in the literature, natural compounds with significant antimycobacterial activity are reported, it is notable that most of the researchers are focusing mainly on evaluation of synthetic drugs. The anti-TB effect of most of the plant compounds has not yet been confirmed in animal experiments. Primary reasons for this are (1) most of the research laboratories do not have animal experimentation facility (BSL-3) for the study of *in vivo* anti-TB effects of phytomolecules (2) difficulty in solubility of the natural product (3) lack of data on pharmacokinetics, pharmacodynamics and toxicological studies (4) unavailability of sufficient amount of the pure compound (5) high cost of carrying out the *in vivo* tests, among others.

Considering the above situation, there is a need to identify the potential of anti-TB phytomolecules having selective toxicity and satisfactory pharmacokinetic and pharmacodynamic parameters in animal TB models. From the above studies, it can be concluded that plants represent new sources of anti-TB agents but only few plant species have been thoroughly investigated for their anti-TB potential and thus there is a renewed opportunity in utilizing phytomedical research for TB drug discovery from alternative sources.
